# An acoustic source model for asymmetric intraglottal flow with application to reduced-order models of the vocal folds

**DOI:** 10.1371/journal.pone.0219914

**Published:** 2019-07-25

**Authors:** Byron D. Erath, Sean D. Peterson, Kelley S. Weiland, Michael W. Plesniak, Matías Zañartu

**Affiliations:** 1 Department of Mechanical and Aeronautical Engineering, Clarkson University, Potsdam, NY, United States of America; 2 Department of Mechanical and Mechatronics Engineering, University of Waterloo, Waterloo, Ontario, Canada; 3 Naval Surface Warfare Center, Dahlgren Division, Dahlgren, VA, United States of America; 4 Department of Mechanical and Aerospace Engineering, The George Washington University, Washington, D.C., United States of America; 5 Department of Electronic Engineering, Universidad Técnica Federico Santa María, Valparaíso, Chile; Delft University of Technology, NETHERLANDS

## Abstract

The complex three-way interaction between airflow, tissue, and sound, for asymmetric vocal fold vibration, is not well understood. Current modeling efforts are not able to explain clinical observations where drastic differences in sound production are often observed, with no noticeable differences in the vocal fold kinematics. To advance this understanding, an acoustical model for voiced sound generation in the presence of asymmetric intraglottal flows is developed. The source model operates in conjunction with a wave reflection analog propagation scheme and an asymmetric flow description within the glottis. To enable comparison with prior work, the source model is evaluated using a well-studied two-mass vocal fold model. The proposed source model is evaluated through acoustic measures of interest, including radiated sound pressure level, maximum flow declination rate, and spectral tilt, and also via its effects on the vocal fold dynamics. The influence of the model, in comparison to the standard symmetric Bernoulli flow description, results in an increased transfer of energy from the fluid to the vocal folds, increased radiated sound pressure level and maximum flow declination rate, and decreased spectral tilt. These differences are most pronounced for asymmetric vocal fold configurations that mimic unilateral paresis and paralysis, where minor kinematic changes can result in significant acoustic and aerodynamic differences. The results illustrate that fluid effects arising from asymmetric glottal flow can play an important role in the acoustics of pathological voiced speech.

## 1 Introduction

Speech production is the result of complex interactions between flow, tissue, and sound, affecting the vocal fold (VF) dynamics, kinematics, aerodynamics, and radiated pressure at the mouth [1]. Approaches describing these interactions have been applied in models of speech production to study normal and pathological speech [2], and have become relevant clinical tools [3–7]. However, very few studies have been devoted to validate model predictions with comprehensive recordings in human subjects. Direct comparison of VF kinematics obtained with laryngeal high-speed videoendoscopy and radiated pressure has shown that current models of speech production are not capable of fully capturing the complexity of the phenomena, especially under asymmetric VF conditions [3, 8]. Similar observations have been made when assessing the perceptual relevance of the model output for both normal [9] and asymmetric VF vibration [10]. Thus, outstanding issues exist for advancing physics-based descriptions of airflow, sound and tissue interactions in symmetric and asymmetric VF vibration.

Various common modeling assumptions need to be revisited, when studying symmetric and asymmetric VF vibration. Each phonatory cycle is characterized by the propagation of a mucosal wave along the medial surfaces of the VFs that produces a phase delay between the motion of the inferior and superior edges. Consequently, the glottis forms a temporally-varying orifice that transitions from a convergent to divergent configuration throughout each cycle. It is the closure of the VFs that produces many of the clinically-relevant indicators of speech quality, such as the speed quotient and maximum flow declination rate [11, 12]. The divergent VF configuration gives rise to the development of rich viscous flow behavior, including unsteady flow separation [13], asymmetric jet behavior [14–19], vortex shedding [20–22], and significant boundary layer growth [23]. The effect of these phenomena on the vibratory characteristics and resulting sound in normal and pathological speech is an important topic of research [24].

Efforts to deduce the impact of asymmetric intraglottal flows on VF dynamics have been performed using computational investigations of fully-coupled fluid-structure interactions [25], combining computational fluid dynamics (CFD) flow solvers with reduced-order VF models [26, 27], and more recently, by developing theoretical flow solutions [28, 29]. A boundary-layer estimation of the asymmetric pressures (BLEAP) to predict the pressure loadings that occur during plane-wall asymmetric intraglottal flow attachment has been developed [28], while a separate approach has considered the physics that arise as the flow follows the glottal inlet radius on one wall, while separating from the opposing wall, thereby asymmetrically skewing the flow within the glottal inlet [29, 30].

Theoretical asymmetric flow solutions applied to low-dimensional models and high-fidelity computational efforts have both demonstrated that asymmetric glottal flow creates amplitude asymmetries in the VF dynamics, becoming most pronounced for high subglottal pressures and when tension imbalances between the VFs are present, which can quickly lead to nonlinear dynamics [26, 31]. The influence of asymmetric behavior on acoustic measures, however, is not so clear. Asymmetric glottal flows are expected to influence not only the loading on the VF structure, and therefore the kinematics, but also the acoustics due to modulation of the dipole sound source arising from the change in pressure losses through the glottis [31]. Consequently, modeling the impact of asymmetric flows on the acoustic source may yield insight into unexplained variance in acoustic measures; i.e., acoustic variations that do not directly correlate with kinematic asymmetries [8, 10].

The objective of this study is to derive and evaluate a new acoustic solver that is consistent with asymmetric glottal flow behavior to assess the impact of asymmetric glottal flow on acoustic outputs. The scheme is based on a time-domain acoustic source model that is extended for asymmetric glottal flow and contrasted with a standard symmetric glottal flow formulation. The effects of the proposed acoustic solver on the kinematics of the VFs and on the resulting sound generation are evaluated using a well-known reduced-order VF model [32] to allow for initial comparisons.

The manuscript is outlined as follows. The derivation of the acoustic model is presented in § 2 and details of the numerical solution and analysis procedures are presented in § 3. Application to a reduced-order speech model, and sensitivity of the acoustic model is discussed in § 4, and § 5 is left for the conclusions.

## 2 Theory

**Fig 1:**
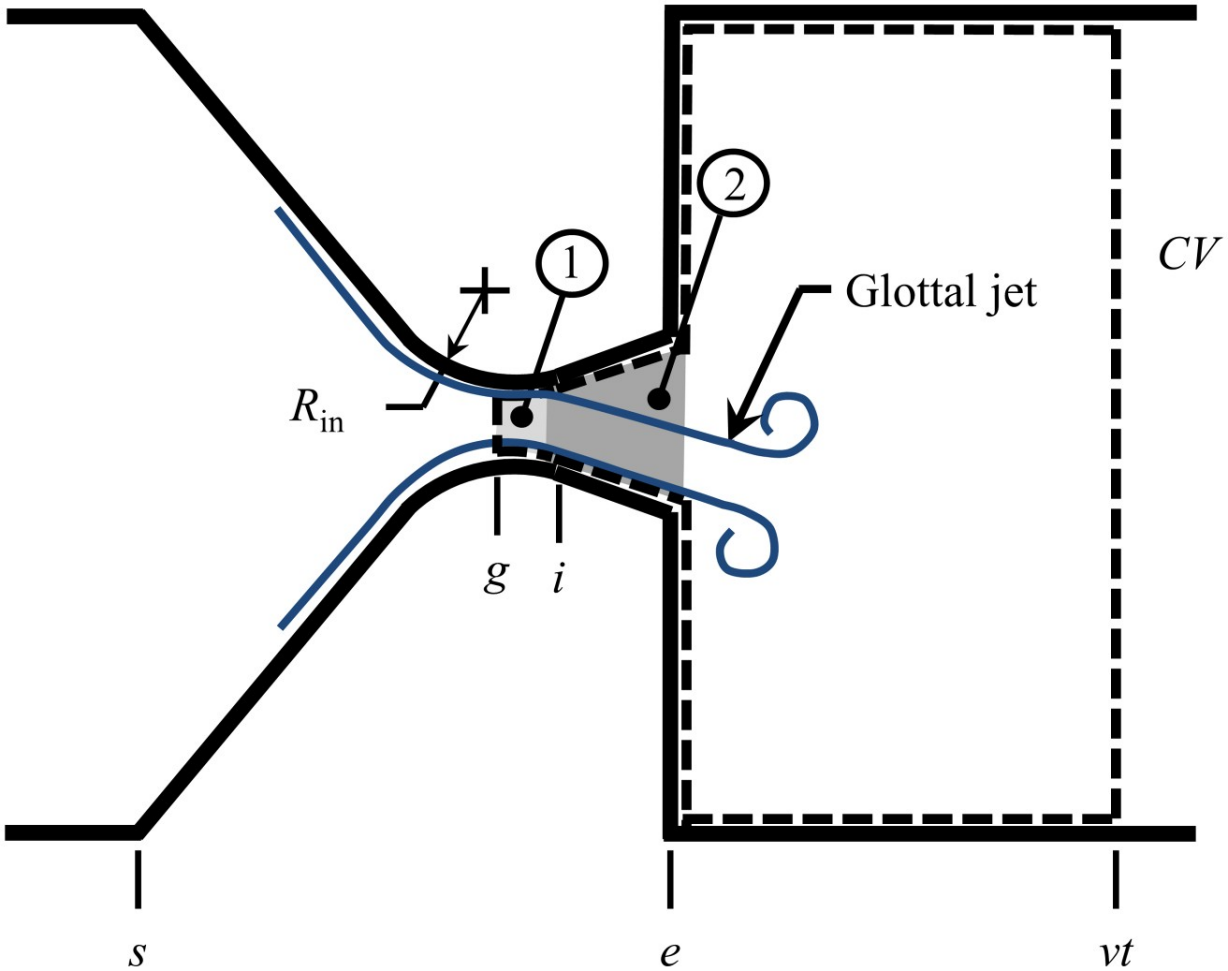
Schematic of the glottal fluid and acoustic model domain. Region (1) denotes where the effects of flow curvature are modeled, while (2) identifies where the Boundary Layer Estimation of the Asymmetric Pressures (BLEAP) model is applied. [28]. Locations of interest are identified as: *s*—subglottal; *g*—glottis, *i*—inlet, *e*—exit, *vt*—vocal tract.

In this study, an *acoustic* source model for asymmetric glottal flow is paired with an asymmetric intraglottal *flow* solver for use in reduced-order VF models [28]. The fluid solver is based on prior modeling efforts that implemented a Boundary Layer Estimation of the Asymmetric Pressures (BLEAP) to determine the effect of asymmetric fluid loading arising from glottal jet asymmetry [28]. This approach is updated to also consider the effects of flow curvature at the inlet to the glottis [29, 30]. The proposed *acoustic* solver is an extension of the work initially proposed by Titze [33, 34], where the primary glottal dipole source was described in the framework of a symmetric Bernoulli flow coupled with a wave reflection analog sound propagation scheme. This new *acoustic* source model is derived using a similar approach, albeit for asymmetric glottal flow orientations, ensuring it is broadly applicable to virtually all structural VF models. The framework of the approach is outlined in Fig 1, where the control volume (CV) used to derive the acoustic sound propagation scheme is indicated. Different regions of the flow field are denoted as number (1) and (2), and discrete locations are denoted by “*s*” for subglottal, “*g*” for glottis, “*i*” for inlet, “*e*” for exit, and “*v*t”, for vocal tract. These abbreviations will subsequently be used as subscripts to denote the location of velocities and pressures.

### 2.1 Flow solution

The following sections outline the solution for determining the fluid loading in regions (1) and (2) that acts on the VFs. When the VFs form a divergent passage, the glottal jet asymmetrically attaches to one VF wall [14, 15, 17, 23, 35]. In region (1), the VF loading due to the flow asymmetrically skewing towards one glottal wall is computed (see Section 2.1.1), while in region (2) the pressure loading is computed for an attached wall jet in an adverse pressure gradient (see Section 2.1.2). The combination of these forces yields the total lateral VF loading arising from the asymmetrically-attached glottal jet. The high velocity jet is assumed inviscid in region (1), but not in region (2) where boundary layer growth is appreciable. Both regions approximate the flow as quasi-steady [36].

#### 2.1.1 Flow curvature

**Fig 2:**
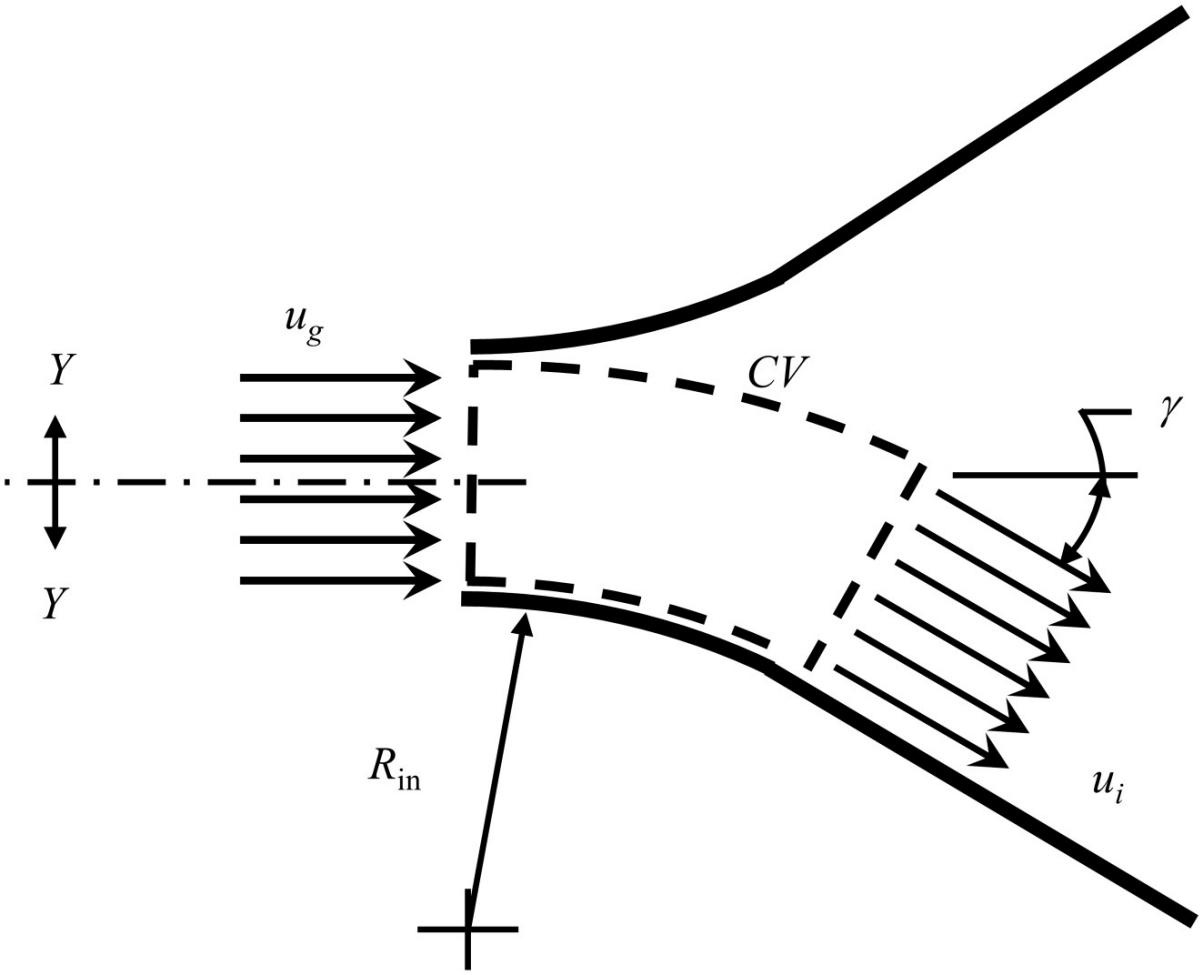
Schematic of the glottal fluid and acoustic model domain. Region (1) denotes where the effects of flow curvature are modeled, while (2) identifies where the Boundary Layer Estimation of the Asymmetric Pressures (BLEAP) model is applied. [28]. Locations of interest are identified as: *s*—subglottal; *g*—glottis, *i*—inlet, *e*—exit, *v*t—vocal tract.

It has been suggested that because the glottal jet asymmetrically bends towards a VF wall, lateral loads arising due to flow curvature should be considered [29, 30]. The pressure loading arising solely due to flow curvature can be computed in region (1) in Fig 1. As the glottal jet follows the inlet radius of the glottis, *R*_in_, and separates from the opposing wall at the minimal glottal area (denoted by subscript “*g*”), the attached flow along the wall is assumed to be inviscid and uniform. Therefore, performing an integral momentum balance on the control volume displayed in Fig 2 shows that the lateral load is balanced by the angular variation in the momentum flux of the glottal jet. The pressure and velocity at the minimal glottal area (the inlet to region (1) in Fig 1) are denoted as *p*_*g*_, and *u*_*g*_, respectively, while the pressure and velocity downstream of the bend (the boundary between regions (1) and (2) in Fig 1) is denoted as *p*_*i*_, and *u*_*i*_, respectively. However, by assuming the flow is inviscid in this region and that the jet area remains constant, *p*_*g*_ = *p*_*i*_, and *u*_*g*_ = *u*_*i*_. Note *γ* is the glottal wall angle, where a positive value for denotes a convergent glottal configuration, and a negative value a divergent configuration.

Note that the original formulation for computing lateral loads from flow curvature [29] assumes that ambient pressure acts on the upper curved surface of the CV in Fig 2, which is not compatible with formulations that include acoustic wave propagation in the supraglottal tract. In the following analysis it is therefore assumed that the pressure at the exit of the glottal jet, *p*_*e*_, acts on the wall from which the flow is separated (non-flow wall), and therefore also acts on the upper curved surface of the CV shown in Fig 2. The reactionary forces on the flow and non-flow walls in region (1) of Fig 1 can then be found by evaluating the momentum fluxes through the CV shown in Fig 2, which are given as x

$$\begin{array}{ccc} {\vec{F}}_{\text{flow}} & = & \left[p_{e}(A_{i}+R_{\text{in}}L)-p_{i}A_{i}-\rho u_{i}^{2}A_{i}\right]\\ & & (1-\text{cos}\;\gamma )\hat{i}\\ & + & [A_{i}+p_{e}(A_{i}p_{i}+R_{\text{in}}L)+\rho u_{i}^{2}A_{i}](\text{sin}\;\gamma )\hat{j}, \end{array}$$ (1)

and

$$\begin{array}{ccc} {\vec{F}}_{\text{no}-\text{flow}} & = & p_{e}R_{\text{in}}L(1-\text{cos}\;\gamma )\hat{i}\\ & + & p_{e}R_{\text{in}}L\;(\text{sin}\;\gamma )\hat{j}, \end{array}$$ (2)

respectively, where *L* is the anterior-posterior length of the VFs, *ρ* is the fluid density, and *A* is the cross-sectional area at corresponding location indicated by the subscript.

#### 2.1.2 BLEAP

To determine the loads acting on the VF walls in region (2) of Fig 1, the previously developed BLEAP approach is implemented. While a brief overview of the BLEAP approach is provided herein, a more detailed analysis can be found in Erath et al. [28].

**Fig 3:**
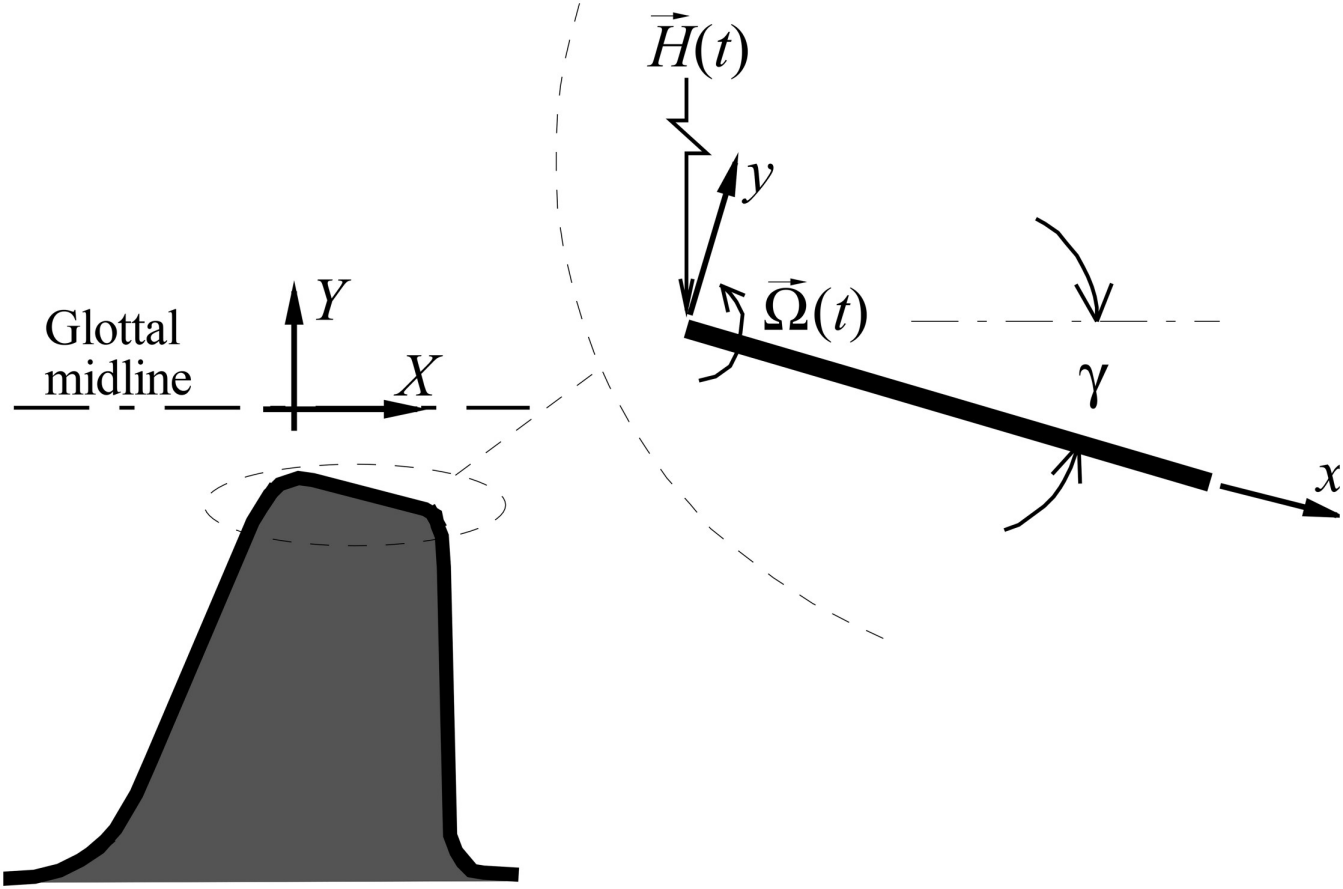
Schematic of a simplified model of vocal fold kinematics. The model vocal fold is represented by a translating and rotating flat plate.

To model asymmetric glottal flow attachment the medial surface of the VF wall to which the flow is attached is approximated as a rotating and translating flat plate (shown in Fig 3) with glottal jet velocity *u*_*g*_ passing over it. Rotation occurs about the leading edge of the plate, defined by the angular velocity $\vec{\Omega }\left(t\right)$, and the translational motion $\vec{H}\left(t\right)$ is constrained in the *Y* direction, where *X*, *Y* denotes an inertial reference frame, and *x*, *y* is a non-inertial reference frame attached to the leading edge of the plate. The plate has thickness *w* in the *z* direction, and the angle of deflection from the *X* direction is given as *γ*, as discussed in Section 2.1.1.

When solved in the non-inertial reference frame the Navier-Stokes equations can be reduced to the standard boundary layer equations in the chosen reference frame, where a similarity variable,

$$\begin{array}{c} \eta =y\sqrt{\frac{u^{{}^{\prime }}}{\beta \nu }} \end{array}$$ (3)

is found, where the prime indicates differentiation with respect to *x*, *ν* is the kinematic viscosity of air, and

$$\begin{array}{c} \beta =\frac{2n}{\left(n+1\right)}. \end{array}$$ (4)

The constant *β* is related to the pressure gradient in the divergent glottis, which arises due to the divergent glottal orientation. As such, *β* is directly related to the glottal divergence angle, and is a function of the experimental parameter, *n*, which is found from the similarity solution. Originally, a constant value of *β* was proposed [28], however, physically it is expected that the pressure gradient within the glottis, and thus *β*, will vary with the included glottal divergence angle. Consistent with the lumped-element modeling approach, the variable *β* can be assumed to vary in a quasi-steady fashion.

From the similarity solution, velocity *u*(*x*) in the inviscid jet core can be expressed as

$$\begin{array}{c} u\left(x\right)=c_{0}{\left(x+x_{\text{off}}\right)}^{n}, \end{array}$$ (5)

where the variable *x*_off_ is an experimentally determined constant to ensure an appropriate boundary layer thickness at the glottal entrance, found to be *x*_off_ = 0.20 mm. The constant *c*_0_ is

$$\begin{array}{c} c_{0}=\frac{u_{i}}{x_{\text{off}}^{n}}, \end{array}$$ (6)

where *u*_*i*_ is the velocity at the inlet to the glottis. Bernoulli’s equation is applied in the core of the glottal jet to solve for the streamwise pressure gradient, which is then imposed on the glottal wall as the wall normal pressure gradient is zero [28]. The utilization of Bernoulli’s equation within the core of the glottal jet is justified by recognizing that the viscous stresses within the core are negligible, as previously shown [1, 30]. Solving for the pressure *p*_*i*_ and velocity at the glottal entrance, the details of which are not shown here, yields

$$\begin{array}{c} p_{i}=p_{s}-\frac{1}{2}\rho u_{i}^{2}, \end{array}$$ (7)

where

$$\begin{array}{c} u_{i}=\sqrt{\frac{2(p_{s}-p_{e})}{\rho }}{\left(\frac{x_{\text{off}}}{x_{e}+x_{\text{off}}}\right)}^{n}, \end{array}$$ (8)

and *p* is pressure, and *x*_*e*_ is the length of the glottal wall in the streamwise direction. Finally, it can be shown that the pressure in the inviscid core of the glottal jet can be imposed on the VF wall to which it is attached, with the distribution given by

$$\begin{array}{c} p\left(x\right)=p_{i}+\frac{1}{2}\rho [u_{i}^{2}-u{\left(x\right)}^{2}+\Omega^{2}{\left(x_{\text{off}}+x\right)}^{2}]. \end{array}$$ (9)

Note the addition of the centripetal acceleration term that was mistakenly omitted in the original solution development. [28]

The attached glottal jet produces asymmetric fluid loading, which can be found as

$$\begin{array}{c} F_{\text{asym},\alpha }\left(t\right)=\int_{0}^{x_{e}}p\left(x\right)dx, \end{array}$$ (10)

where *p*(*x*) is given by Eq 9. The subscript *α* denotes the wall to which the flow is attached, with *α* = *L* or *R*, indicating the left or right VF, respectively. Based upon experimental evidence [14, 16, 17], the flow attaches to the wall with the shallower divergence angle, *γ*. Consequently, the glottal jet attachment can change on a cycle-to-cycle basis, as determined by the glottal geometry.

**Fig 4:**
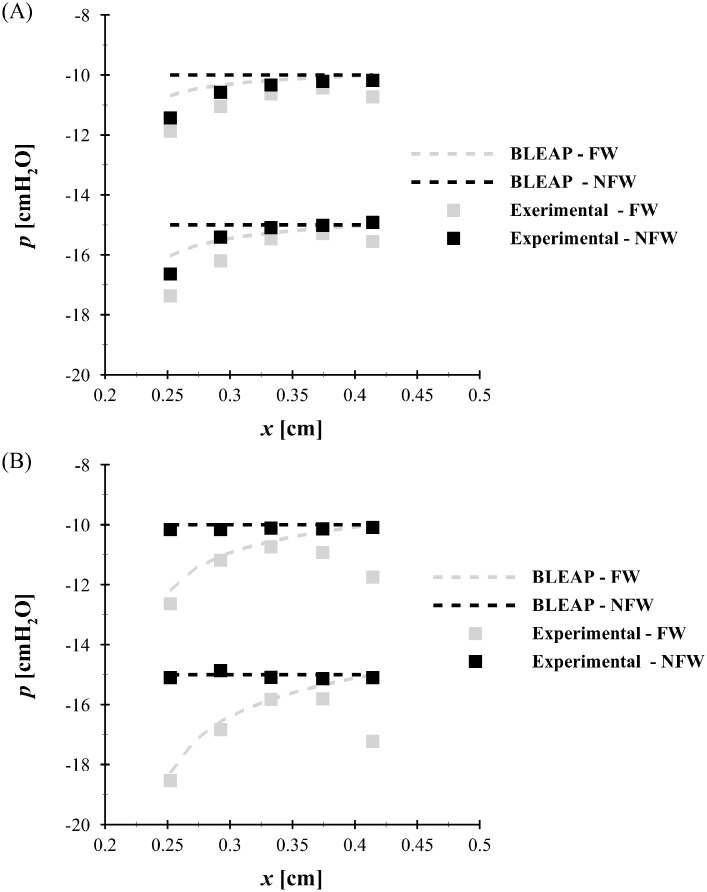
Intraglottal wall pressures. Experimental (■) and intraglottal pressure estimation predicted by the BLEAP flow solver (- -) for two transglottal pressure drops of 10 cmH_2_O and 15 cmH_2_O, where black color denotes the pressure on the non-flow wall, and gray color denotes the pressure on the flow wall. Data are presented for A: an included glottal divergence angle of 10° with *n* = −0.015, and B: an included glottal divergence angle of 40° with *n* = −0.05.

The accuracy of the BLEAP approach is validated by comparing it with the intraglottal pressure measurements acquired in the ubiquitous M5 geometry [37], shown in Fig 4 at two transglottal pressure drops of 10 cmH_2_O and 15 cmH_2_O. Note that only the pressure along the medial surface of the vocal folds is reported, as that is the location that is modeled by the BLEAP scheme. The data presented in Fig 4A are for an included divergence angle of 10° [37], with an exponent value of *n* = −0.015, as originally proposed [28]. In Fig 4B the experimental data [38] are shown for an included divergence angle of 40°, where the exponent of the BLEAP solution was empirically chosen to fit the experimental measures of intraglottal pressure, resulting in a value of *n* = −0.05. Note that as the axial position of the pressure location (*x*) increases, the experimental pressure measured along the flow wall reaches a maximum, and then decreases. This secondary drop in the pressure arises due to centripetal acceleration of the flow as it follows the contour of the M5 geometry around a radius at the glottal exit. As this exit radius is not considered in the BLEAP formulation (Fig 3), this effect is not captured.

Nevertheless, there is good agreement between the experimentally-measured pressures, and those predicted by the BLEAP formulation. For lower divergence angles Fig 4A, a pressure recovery is observed on both walls in the experimental data, although this is only predicted for the flow wall in the BLEAP formulation. The difference in the pressure between the opposing walls, which is what governs the physics of the reduced-order model oscillations, is, however, well predicted. For higher divergence angles Fig 4B, very good agreement is found for the BLEAP solution. Because experimental investigations have shown that for divergence angles greater than 40°, the flow fully-separates from VF walls, based on these observations, a value of *n* = −0.05 is chosen as an upper limit for the subsequent investigations that consider a temporally-varying *n* value.

### 2.2 Wave-reflection analog

To model the influence of asymmetric flow on the acoustics, sound propagation in the subglottal and supraglottal tract is solved using the wave reflection analog (WRA) approach [39–41]. Each vocal tract area function is discretized as multiple short uniform tubes, with reflection coefficients at each junction. A resistive lung termination and inertive radiation impedance at the lips is also included.

The WRA formulation proceeds as specified by Titze [33], with the exception that the volumetric flow rate *Q* is computed using the asymmetric flow solution outlined in Section 2.1, as opposed to a symmetric Bernoulli solver, as is traditionally implemented [33].

A brief overview of the WRA solution method follows. The pressures in the subglottal (*p*_*s*_) and supraglottal (vocal tract) (*p*_*vt*_) regions can be expressed as the superposition of forward and backward traveling waves, denoted by superscript (+) and superscript (−), respectively, as

$$\begin{array}{c} p_{s}=p_{s}^{+}+p_{s}^{-}, \end{array}$$ (11)

$$\begin{array}{c} p_{vt}=p_{vt}^{+}+p_{vt}^{-}. \end{array}$$ (12)

Conservation of mass yields two additional equations, given by

$$\begin{array}{c} Q=\frac{A_{s}}{\rho c}(p_{s}^{+}-p_{s}^{-}), \end{array}$$ (13)

$$\begin{array}{c} Q=\frac{A_{vt}}{\rho c}(p_{vt}^{+}-p_{vt}^{-}). \end{array}$$ (14)

Combining Eqs 11 and 12 with Eqs 13 and 14, yields

$$\begin{array}{c} p_{s}=2p_{s}^{+}-\frac{\rho cQ}{A_{s}}, \end{array}$$ (15)

$$\begin{array}{c} p_{vt}=2p_{vt}^{-}+\frac{\rho cQ}{A_{vt}}, \end{array}$$ (16)

The pressures *p*_*s*_ and *p*_*vt*_ are specified a priori, from which the outgoing pressures, $p_{s}^{-}$ and $p_{vt}^{+}$ are determined using Eqs 11 and 12, because $p_{s}^{+}$ and $p_{vt}^{-}$ are both initially 0.

With the pressures known at the zeroth time step, the wave propagation scheme that tracks the transmission and reflection of the pressure waves at each junction in the subglottal and supraglottal tract is utilized to find the pressure waves incident on the glottis ($p_{s}^{+}$ and $p_{vt}^{-}$), where the acoustic flow rate *Q*, is computed from the chosen acoustic flow solver. In Section 2.3 a new asymmetric acoustic flow solver will be derived. In all cases, the acoustic flow rate is found using $p_{s}^{+}$ and $p_{vt}^{-}$ as inputs, as well as the glottal area, *A*_*i*_, at the corresponding time. The acoustic flow rate and the pressures incident on the VFs ($p_{s}^{+}$ and $p_{vt}^{-}$) are then input into Eq’s 13 and 14 to solve for the new outgoing pressure waves ($p_{s}^{-}$ and $p_{vt}^{+}$) from the VFs, where the volumetric flow rate, *Q*, is prescribed by the chosen symmetric or asymmetric solution. These outgoing pressures ($p_{s}^{-}$ and $p_{vt}^{+}$) are then input into the wave propagation scheme at the next time step, and the process is repeated.

### 2.3 BLAST solver

The traditional WRA acoustic solution for speech uses a symmetric, inviscid flow solver (Bernoulli’s equation) to prescribe the flow through the VFs [33]. When coupling the WRA solver for acoustic wave propagation with the curvature and BLEAP flow solvers, it is necessary to re-derive the WRA solution under the assumption of asymmetric flow in order to maintain physical consistency between the fluid and acoustic solvers. This updated WRA acoustic solution for asymmetric intraglottal flow will be referred to as the Boundary Layer Acoustic Source Term (BLAST) solver. The derivation of the BLAST solver follows.

Conservation of linear momentum in the *X* direction for a control volume spanning from the glottis (“*g*”) to a downstream location in the vocal tract (“*vt*”), as denoted in Fig 1, reduces to

$$\begin{array}{c} \sum F_{X}=\int_{CS}\rho u\vec{V}\cdot d\vec{A}, \end{array}$$ (17)

where *u* is the velocity component in the *X* direction, and $\vec{A}$ is a directed, outward-pointing area. This control volume formulation is very similar to that employed by Titze [33] in the original derivation of his Bernoulli-WRA method. The only deviation from Titze’s control volume configuration is the location of the upstream entrance to the control volume, which is herein placed at the minimal glottal diameter, as opposed to the glottal exit. Following Titze [33], this formulation assumes that the temporal variation of linear momentum within the control volume is small in comparison with the momentum flux. The finite distance between the glottal exit (“*e*” in Fig 1) and the beginning of the vocal tract (“*vt*”) in Fig 1 assumes losses due to mixing as the glottal jet expands to fill the vocal tract are small, and hence, the velocity at “*vt*” can be assumed uniform. Neglecting gravity and viscous terms, the only forces present are surface forces arising due to the pressure; hence the left side of the equation becomes

$$\begin{array}{ccc} \sum F_{X} & = & p_{i}A_{i}-p_{vt}A_{vt}+p_{e}\left(A_{vt}-A_{e}\right)\\ & + & {\vec{F}}_{\text{flow}}\cdot \hat{i}+{\vec{F}}_{\text{no}-\text{flow}}\cdot \hat{i}\\ & + & \frac{1}{2}p_{e}\left(A_{e}-A_{i}\right)+w\int_{0}^{x_{e}}p\left(x\right)\;\text{sin}\;\gamma dx, \end{array}$$ (18)

where *A*_*i*_ is the inlet area, *A*_*vt*_ is the vocal tract area at the downstream end of the control volume, *A*_*e*_ is the area at the exit of the glottis, and *F*_flow_ and *F*_no−flow_ are given by Eqs 1 and 2, respectively. Viscous terms are neglected, as prior analysis (not shown here for brevity) reveals they are several orders of magnitude smaller than the remaining terms, producing errors of less than 1%. In Eq 18 it is further assumed that *p*_*e*_ acts on the non-flow wall (as assumed in the BLEAP flow derivation), and the pressure on the flow wall can be found from Eq 9, which can be expressed in terms of *Q* as

$$\begin{array}{c} p\left(x\right)=p_{s}-\frac{1}{2}\rho {\left(\frac{Q}{A_{i}}\right)}^{2}{\left(\frac{x+x_{\text{off}}}{x_{\text{off}}}\right)}^{2n}. \end{array}$$ (19)

Applying Bernoulli’s equation in the subglottal tract, where it is valid due to the favorable pressure gradient, and recognizing that due to the inviscid assumption of the flow curvature *p*_*g*_ = *p*_*i*_, allows the pressure at the inlet to the straight-walled portion of the diffuser to be related to the glottal exit pressure via Eq 9, which results in equations for the pressures at *p*_*i*_ and *p*_*e*_ in terms of known quantities,

$$\begin{array}{c} p_{i}=p_{s}-\frac{1}{2}\rho {\left(\frac{Q}{A_{i}}\right)}^{2}, \end{array}$$ (20)

$$\begin{array}{c} p_{e}=p_{s}-\frac{1}{2}\rho {\left(\frac{Q}{A_{i}}\right)}^{2}\Gamma^{2n}+\frac{1}{2}\rho \Omega^{2}{\left(x_{e}+x_{\text{off}}\right)}^{2}, \end{array}$$ (21)

where Γ = (*x*_*e*_ + *x*_off_)/*x*_off_. Using the fact that *x*_*e*_*w* sin *γ* = (1/2)(*A*_*e*_ − *A*_*i*_) Eq 18 can then be simplified to

$$\begin{array}{ccc} \sum F_{x} & = & p_{s}[A_{vt}+2R_{\text{in}}w(1-\text{cos}\;\gamma )]-p_{vt}A_{vt}\\ & - & \frac{1}{2}{\left(\frac{Q}{A_{i}}\right)}^{2}\{A_{vt}\\ & & -\frac{1}{2}[A_{e}-A_{i}(1-2\;\text{cos}\;\gamma )]\\ & & +2R_{in}w\;(1-\text{cos}\;\gamma )\}\\ & + & \frac{1}{2}(A_{i}-A_{e})\frac{1}{2n+1}(\frac{x_{\text{off}}}{x_{e}})[1-\Gamma^{2n+1}]\\ & + & A_{i}(2-\text{cos}\;\gamma )\\ & - & \frac{1}{2}\rho \Omega^{2}{\left(x_{\text{off}}+x_{e}\right)}^{2}\\ & & \left\{A_{vt}-\frac{1}{2}[A_{e}-A_{i}(1-2\;\text{cos}\;\gamma )\right]\\ & & +2R_{in}w\;(1-\text{cos}\;\gamma )\}\\ & + & \frac{1}{12}(A_{e}-A_{i})\{(\frac{1}{x_{e}})\\ & & \rho \Omega^{2}[{\left(x_{\text{off}}+x_{e}\right)}^{3}-x_{\text{off}}^{3}]\}. \end{array}$$ (22)

Returning now to Eq 17, the right hand side can be expressed as

$$\begin{array}{c} \int_{CS}\rho u\vec{V}\cdot d\vec{A}=-\rho {\left(\frac{Q}{A_{i}}\right)}^{2}A_{i}(1-\frac{A_{i}}{A_{vt}}). \end{array}$$ (23)

Substituting Eqs 22 and 23 into Eq 17, dividing through by *A*_*i*_, and simplifying with laborious algebra produces

$$\begin{array}{ccc} & & \left(\frac{A_{vt}}{A_{i}})\{p_{s}[1+\frac{2R_{\text{in}}w\;(1-\text{cos}\;\gamma )}{A_{vt}}]-p_{vt}\right\}\\ & = & \frac{1}{2}\rho {\left(\frac{Q}{A_{i}}\right)}^{2}\Pi_{1}+\Pi_{2}, \end{array}$$ (24)

where

$$\begin{array}{ccc} \Pi_{1} & = & \Gamma^{2n}[\frac{A_{vt}}{A_{i}}-\frac{1}{2}\frac{A_{e}}{A_{i}}+\frac{1}{2}-\text{cos}\;\gamma \\ & & +\frac{2R_{\text{in}}w\;(1-\text{cos}\;\gamma )}{A_{i}}]\\ & + & 2\frac{A_{i}}{A_{vt}}-\text{cos}\;\gamma \\ & + & \frac{1}{2}(1-\frac{A_{e}}{A_{i}})[\frac{1}{2n+1}\frac{x_{\text{off}}}{x_{e}}(1-\Gamma^{2n+1})], \end{array}$$ (25)

and

$$\begin{array}{ccc} \Pi_{2} & = & \frac{1}{2}\rho \Omega^{2}{\left(x_{\text{off}}+x_{e}\right)}^{2}\\ & & [\frac{A_{v}t}{A_{i}}-\frac{1}{2}\frac{A_{e}}{A_{i}}+\frac{1}{2}-\text{cos}\;\gamma \\ & & +\frac{2R_{\text{in}}w\;(1-\text{cos}\;\gamma )}{A_{i}}]\\ & + & \frac{1}{12}(\frac{1}{x_{e}})(1-\frac{A_{e}}{A_{i}})\rho \Omega^{2}\\ & & [{\left(x_{\text{off}}+x_{e}\right)}^{3}-x_{\text{off}}^{3}]. \end{array}$$ (26)

After substituting for *p*_*s*_ and *p*_*vt*_ from Eqs 15 and 16, the final expression for the volumetric flow rate *Q* arising from asymmetric intraglottal flow is found to be

$$\begin{array}{ccc} & & \frac{1}{2}\rho \Pi_{1}{\left(\frac{Q}{A_{i}}\right)}^{2}\\ & + & \rho c(\frac{Q}{A_{i}})[\frac{A_{vt}}{A_{s}}+\frac{2R_{\text{in}}w\;(1-\text{cos}\;\gamma )}{A_{s}}+1]\\ & - & 2\frac{A_{vt}}{A_{i}}\{p_{s}^{+}[1+\frac{2R_{\text{in}}w\;(1-\text{cos}\;\gamma )}{A_{vt}}]-p_{vt}^{+}\}\\ & + & \Pi_{2}=0. \end{array}$$ (27)

Eq 27 is a quadratic equation in *Q* that yields two possible solutions. The positive value for the radical should be used when $p_{s}^{+}>p_{vt}^{-}$, and the negative value when $p_{s}^{+}<p_{vt}^{-}$. Thus, the BLAST solver takes $p_{s}^{+}$ and $p_{vt}^{-}$ as input and yields *Q* as output, from which the reflected pressures $p_{s}^{-}$ and $p_{vt}^{+}$ can be computed from Eqs 13 and 14. The solution provides inverted pressures for the subglottal tract with respect to the supraglottal tract, which describes the dipole nature of the acoustic source.

### 2.4 Reduced-order vocal fold model

To quantify the effect of intraglottal flow asymmetry on the acoustic output of voiced speech, the aforementioned flow and acoustic equations are implemented into a reduced-order, two-mass VF model, which follows the approach of Steinecke and Herzel (SH) [32]. The SH model is briefly described here for consistency. It is emphasized that the BLAST solver is, however, widely applicable to reduced-order VF models. The SH model represents each VF as two coupled spring-mass-dampers. Model parameters are specified using two subscripts, *j*, and *α*. The inferior and superior masses are denoted by the subscript *j* = 1 and 2, respectively, and *α* = *L* and *R* indicates the left and right VF, respectively, as discussed in § 2.1.2. Masses are represented as *m*_*jα*_, spring constants are *k*_*jα*_, and damping constants are *b*_*jα*_. A coupling spring connects the two masses on each side and is expressed as *k*_*cα*_, where *c* is a dummy index to distinguish it from the standard spring constant. Finally, a collision spring *c*_*jα*_ models the collision force acting on the VFs when they close. A Heaviside function Θ modulates the collision spring so that it is only activated when the VFs are closed. The values of the lumped-element parameters in the VF model are the same as those specified by SH [32].

The governing equations that determine the VF motion can then be expressed as

$$\begin{array}{c} m_{1\alpha }{\ddot{Y}}_{1\alpha }+b_{1\alpha }{\dot{Y}}_{1\alpha }+k_{1\alpha }Y_{1\alpha }+\Theta \left(-a_{1}\right)\frac{c_{1\alpha }a_{1}}{2l}\\ \;+k_{c\alpha }\left(Y_{1\alpha }-Y_{2\alpha }\right)=G\left(t\right) \end{array}$$ (28a)

$$\begin{array}{c} m_{2\alpha }{\ddot{Y}}_{2\alpha }+b_{2\alpha }{\dot{Y}}_{2\alpha }+k_{2\alpha }Y_{2\alpha }+\Theta \left(-a_{2}\right)\frac{c_{2\alpha }a_{2}}{2l}\\ \;+k_{c\alpha }\left(Y_{2\alpha }-Y_{1\alpha }\right)=0, \end{array}$$ (28b)

where *Y*_*jα*_ is the displacement of the mass from the glottal midline. Simulations were run for 600 ms using a sampling frequency of *f*_*s*_ = 70.0 kHz. The forcing function *G*(*t*), discussed in § 2.1.2, is computed as the sum of the curvature and BLEAP forces for the flow and non-flow walls as

$$\begin{array}{ccc} G_{\text{flow}}\left(t\right) & = & \int_{0}^{x_{e}}p\left(x\right)\;\text{cos}\;\gamma dx\\ & + & \text{sin}\;\gamma \;[p_{i}A_{i}+p_{e}(A_{i}+R_{\text{in}}L)+\rho u_{i}^{2}A_{i}] \end{array}$$ (29)

and

$$\begin{array}{ccc} G_{\text{no}-\text{flow}}\left(t\right) & = & p_{e}x_{e}\;\text{cos}\;\gamma +p_{e}R_{\text{in}}L\;\text{sin}\;\gamma , \end{array}$$ (30)

respectively.

## 3 Methods

Four independent fluid and acoustic solvers are referred to in this work: 1) The standard Bernoulli fluid solver, which prescribes flow separation at the minimal glottal area [32, 42], coupled with a symmetric WRA acoustic solver [33] 2) the BLEAP fluid solver [28] coupled with a symmetric WRA acoustic solver, 3) the BLAST fluid and acoustic solver with a constant *n* value, and 4) the BLAST fluid and acoustic solver with a varying *n* value (see Section 3.1). These four solvers are displayed in Table 1 for reference. A case number is provided for each solver and each fluid and acoustic solver is defined according to the corresponding intraglottal flow orientation (symmetric or asymmetric). Cases 3 and 4 incorporate the newly derived formulations for acoustic sound propagation with an asymmetric flow configuration, as outlined in Section 2.3. Comparisons between Cases 3 and 4 and Cases 1 and 2 will provide insight into how the new formulation impacts acoustic sound propagation. In all investigations, the supraglottal tract geometry is specified using area functions from physiologically-acquired 3D magnetic resonance imaging (MRI) data for a vowel /a/ phoneme [43]. The subglottal area function is adapted from respiratory system measurements of human cadavers [44] and includes the trachea, bronchi, and a resistive termination impedance (zeroth and first airway generations) [45]. The WRA scheme, which is the foundation for all of the acoustic solvers, includes a mouth radiation impedance and different loss factors for the subglottal and supraglottal tracts [45]. Level 2 interactions, where it is assumed the acoustic pressure is coupled with the static pressure that drives the flow, are investigated [1].

**Table 1:** Flow and acoustic solver reference table. Each of the fluid and acoustic solvers utilized for the investigations is listed, including whether they are based on a symmetric or asymmetric flow formulation. For methods utilizing an acoustic solver, the level of interaction is listed, and for methods utilizing the BLEAP or BLAST solver the value for the flow exponent *n* (constant or varying) is specified.

Case	Model	Flow Solver	Acoustic Solver	Interaction Level	*n* Value
1	Bernoulli-WRA	Symmetric	Symmetric	“2”	N.A.
2	BLEAP-WRA	Asymmetric	Symmetric	“2”	Constant
3	BLAST	Asymmetric	Asymmetric	“2”	Constant
4	BLAST-*n*(*t*)	Asymmetric	Asymmetric	“2”	Varying

### 3.1 Intraglottal pressure gradient

As previously mentioned, results will be presented for both a constant value of *n* = −0.01478 (BLAST (Case 3)), as initially proposed [28], as well as a variable value of *n*, that is directly related to the total included divergence angle.

The solution with varying *n* value is identical to the BLAST (Case 3) solver, except *n* varies in both the flow and acoustic solutions. Consequently, this solver will be referred to as BLEAP-*n*(*t*) (Case 4). A physical argument can easily be made as to why *n* (see Eq 4) should vary with the included glottal divergence angle. The variable *β* can be physically interpreted as a wedge angle along a wall that is angled relative to the incoming flow direction. This angle is directly related to the pressure gradient produced by the flow. Positive values of *n* correspond to wall angles that impinge into the flow creating a favorable pressure gradient, while negative values of *n* are directly related to a wall angle diverging away from the flow direction, thereby creating an adverse pressure gradient.

For the variable exponent investigations, the exponent *n* is linearly related to the total included divergence angle 2*γ*, and is allowed to vary linearly over the range of 0 ≤ *n* < −0.05 as 2*γ* varies between 0° ≤ 2*γ* < −40°. That is to say when *γ* = 0°, the flow solution is approximated by flow over a flat plate, and as the divergence angle becomes more negative, the pressure gradient becomes more adverse until the point at which the flow separates (−40°). A conservative value of −40° was chosen as the angle at which the flow would fully-separate from both walls based upon experimental observations [14, 15] showing that the glottal flow regime transitioned from an attached wall jet to a fully-separated jet for 2*γ* ≤∼ 35° − 40°. The value of *n* at the lower limit is chosen based on the empirical fit of the BLEAP solution to the experimental pressure data presented in Fig 4 for an included divergence angle of 40°.

### 3.2 Asymmetrically-tensioned vocal folds

It is also of interest to consider irregularly tensioned VFs. Recent investigations have shown that the combination of asymmetric fluid loading with irregularly tensioned VFs (with no acoustic loading) incites important changes in the chaotic behavior of the VF dynamics [31]. Utilizing the formulation of SH [32] for superior laryngeal nerve paralysis, the parameters of the right VF parameters are modified relative to the left VF parameters according to

$$\begin{array}{ccc} & m_{j,R} & =m_{j,L}/Z,\;k_{j,R}=Zk_{j,L},\\ & k_{c,R} & =Zk_{c,L},\;c_{j,R}=Zc_{j,L}, \end{array}$$ (31)

where *Z* is a symmetry parameter that can vary as 0 < *Z* < 1.0, with *Z* = 1 producing symmetrically-tensioned VF parameters. Note that the contact dynamics of the original SH model are incorrect for *Z* ≠ 1 [46], however, and the corrected formulation is used herein.

An estimate of the impact of the flow and acoustic solutions on the dynamics is made by computing the right-left oscillation ratios of VF dynamics. This ratio is computed by first finding the fundamental period of oscillation *T* from the time history of *a*_min_. The number of peaks in *m*_1*R*_ and *m*_1*L*_ over the VF cycle period *T* is then determined, and reported as *ϕ*_*R*_ and *ϕ*_*L*_, respectively. The oscillation ratio, Φ, is then computed as

$$\begin{array}{c} \Phi =\{\begin{array}{cc} \frac{\phi_{1R}}{\phi_{1L}} & \text{for}\;\frac{\phi_{1R}}{\phi_{1L}}<1.0\\ \phi_{1R} & \text{for}\;\frac{\phi_{1R}}{\phi_{1L}}=1.0\\ \text{NRP} & \text{for}\;\text{for}\;\;\text{no}\;\;\text{repeating}\;\;\text{pattern} \end{array} \end{array}$$ (32)

The locations of NRP indicate regions of long transients where no repeating pattern was found in *a*_min_. While regime maps of this type have been reported in the literature [31, 32], none have considered the influence of acoustic loading.

## 4 Results

### 4.1 Asymmetric flow and acoustic solver: Influence on vocal fold dynamics

**Fig 5:**
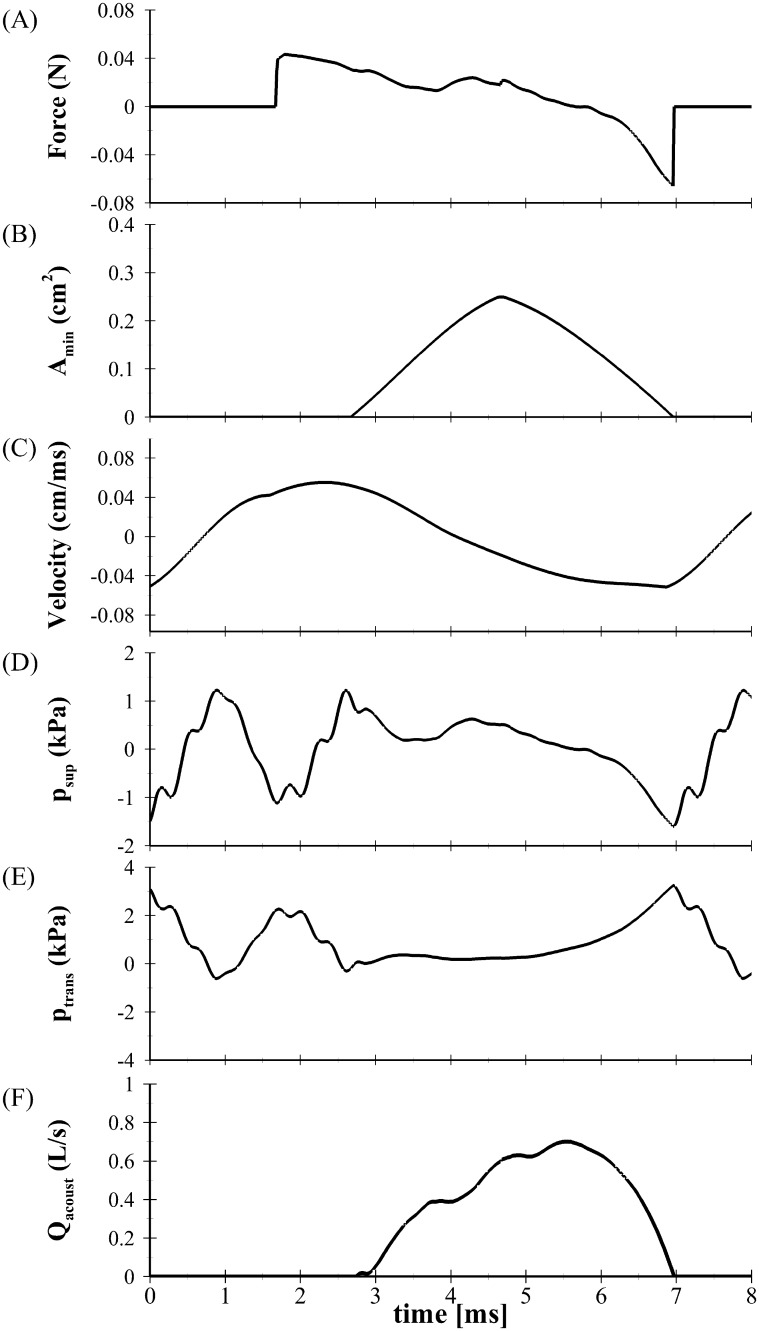
One oscillatory cycle of the aerodynamic parameters produced with the BLAST (case 3) solver. The solution is provided for *p*_*s*_ = 1.0 kPa and *Z* = 1.0. A: fluid loading, B: minimum glottal area, C: velocity of mass 1, D: supraglottal pressure, E: transglottal pressure, and F: flow rate.

To quantify the impact of the BLAST (Case 3) solver, a plot of the fluid load, velocity, minimum glottal area, supraglottal and transglottal pressures, and flow rate is presented in Fig 5A through Fig 5E, respectively. The fluid loading and velocity are presented for the left VF. The lung pressure was specified as 1.0 kPa. As the lower masses (mass 1) of the VFs open (*t* = ∼ 1.7 ms) the subglottal pressure applies a load, forcing them apart, although there is still glottal closure as the upper masses (mass 2) are still obstructing the flow. Glottal opening occurs at ∼ 2.6 ms (Fig 5B). As the flow accelerates through the glottis, the pressure decreases and the loading decreases accordingly. As the VFs begin to close (∼ 4.7 ms) the loading becomes negative. This arises due to (1) the asymmetric fluid solver, which produces a negative glottal gage pressure due to flow curvature and pressure recovery that occurs downstream of the mininal glottal area, and (2) rarefaction of the acoustic pressure in the subglottal tract (Fig 5D through Fig 5E). Because the loading is in phase with the velocity, the fluid imparts energy to the VFs, aiding in closure. The flow rate is in-phase with the glottal opening. While the glottal area is largely symmetric in opening, the flow rate exhibits pronounced skewing as well as ripples in the waveform during opening. These observations are consistent with acoustic loading effects, as previously identified by Zañartu *et. al*., [45].

**Fig 6:**
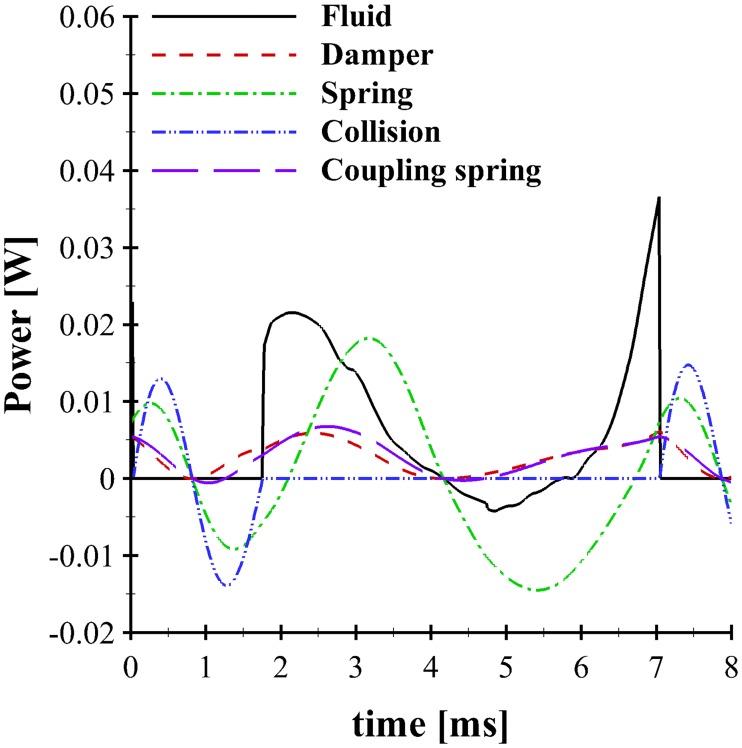
Vocal Fold energy exchange using the BLAST (Case 5) solver. Transfer of energy between the components of mass 1 of the left VF, including the fluid (-), damper (- -), spring (- . -), collision force (- . . -) and coupling spring (– –). The subglottal pressure and symmetry parameter were *p*_*s*_ = 1.0 kPa and *Z* = 1.0, respectively.

A plot of the energy contribution of each component of mass 1 in the VF system is shown in Fig 6. Note that the system is defined as the mass, springs, and dampers of mass 1, such that a positive sign indicates the system absorbing energy. Since the fluid is not part of the system, the positive sign for the fluid indicates energy is imparted to the system, albeit with the aforementioned sign intricacies. The total fluid power transferred to the VF can be computed as the dot product of the force due to the fluid pressure and the velocity from Fig 5A and 5C. Hence, the power is positive when the force and velocity are in the same direction and is negative when they act in opposing directions. For the majority of the cycle, the power is positive, indicating the transfer of energy from the fluid to the system. As the masses begin to open at the start of the cycle (*t* = ∼ 1.7 ms), the first peak in the power curve shows an increased transfer of energy to the mass, facilitated by the concomitant velocity that is observed in Fig 5C. As mass 1 reaches maximum opening and begins to close, energy is transferred back from the system to the fluid for a short period. This occurs as mass 1 is closing, but the total glottal configuration is still convergent (mass 2 is still opening). Later in the cycle (*t* = ∼ 6 ms) the sign of the power changes, indicating the fluid again begins to impart energy to the system. As the VFs begin to close *t* = ∼ 7.0 ms, there is a sharp increase in the amount of power applied to mass 1, as previously discussed. The damper serves only to absorb energy, as is noted by its cyclical pattern that is always positive, and in-phase with the velocity of mass 1. Interestingly, the coupling spring, which attaches mass 1 with mass 2, also serves to act much like a damper, with the second follower mass only absorbing energy from the system. The energy transfer from the spring that connects mass 1 to the ground shows two peaks, with the highest occurring shortly after the masses reach maximum opening. The collision spring, which only activates during VF closure acts to first absorb energy as the VFs contact, and then imparts energy back to the system as the VFs begin to rebound, preceding opening.

**Fig 7:**
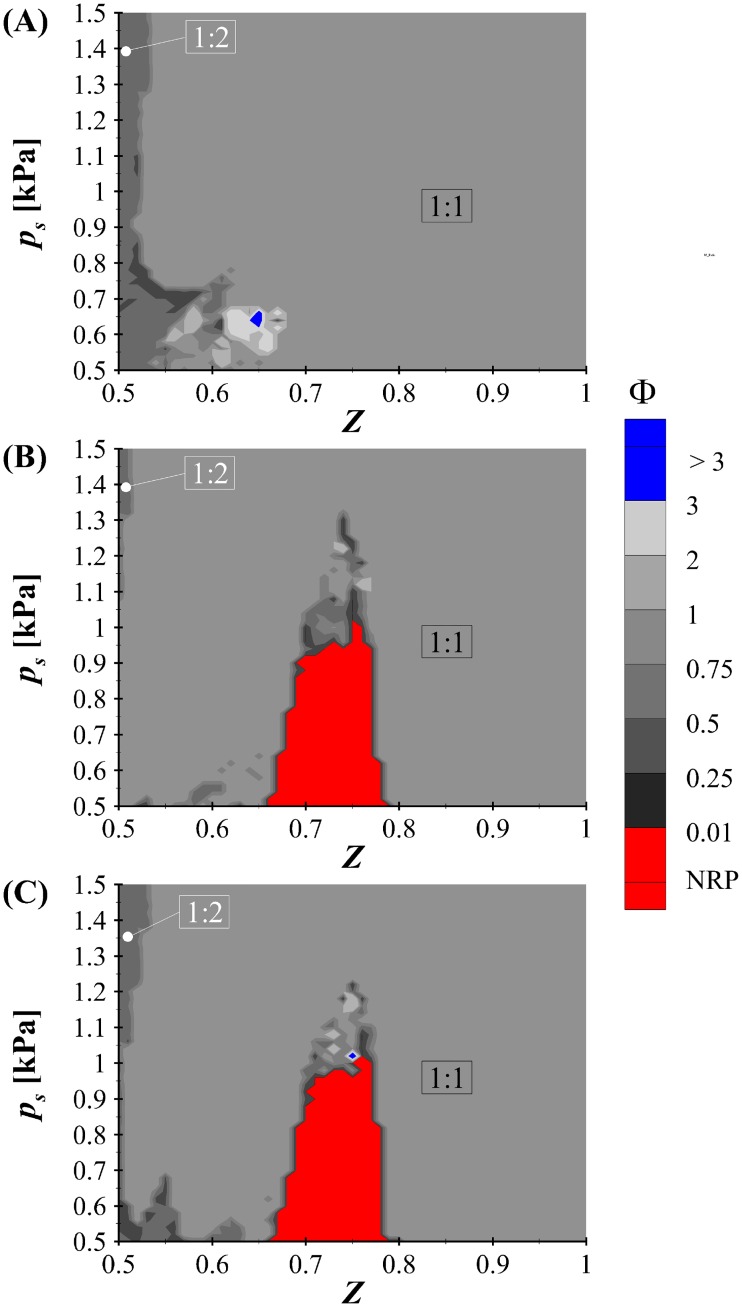
Regime map of the oscillation ratio. Φ as a function of subglottal pressure, *p*_*s*_, and symmetry parameter, *Z*, for the, A: Bernoulli-WRA (Case 1) solver, B: BLEAP-WRA (Case 2) solver, and C: BLAST (Case 3) solver. Regions denoted as no repeating pattern (NRP) denote cases for which self-sustained oscillations were not achieved.

The influence of the BLAST solver on the VF kinematics is investigated by plotting a regime map of the oscillation ratio Φ as a function of subglottal pressure, *p*_*s*_ and symmetry parameter, *Z* as shown in Fig 7. Fig 7A is produced with the Bernoulli-WRA flow and acoustic solver (Case1), Fig 7B employs the BLEAP-WRA flow and acoustic solver (Case 2), while Fig 7C is computed using the BLAST (Case 3) flow and acoustic solver, with constant *n* = −0.01478. Both maps incorporate level 2 interactions and have a resolution of 51 points in both the *p*_*s*_ and *Z* directions. The Bernoulli-WRA solver (Case 1) is included for reference as this is the most ubiquitous solver utilized in lumped-element vocal fold investigations.

Note, as previously discussed, the Bernoulli-WRA solver (Case 1) includes the corrected equations for the collision forces, [46]. As such, the regime does not exhibit the range of rich dynamics that characterized the initial investigations of SH [32], where multiple regimes of fractional oscillation ratios (e.g. 5:8, 3:5, 4:6, etc.), were observed. [32] Nevertheless, there is still a region spanning the lower values of subglottal pressure and asymmetry, where fractional oscillation regimes are evident.

Fig 7B (Case 2) exhibits a marked change in dynamical behavior due to the addition of acoustics when compared to prior investigations of the influence of the BLEAP flow solver with no acoustic interaction [31]. In particular, the variation in oscillation ratios as a function of subglottal pressure and asymmetry parameter is severely diminished. Whereas prior studies of the BLEAP flow solution with no acoustical loading demonstrated large expanses of nonlinear and choatic behavior within the regime map [28, 31] the current BLEAP regime with a symmetric WRA acoustic solver appears to cause more coupled oscillations, with most regions falling within the ratio of 1:1, with the exception of a narrow band of 1:2 at very low asymmetry values. Of note, a relatively large region also exists spanning ∼0.65 < *Z* <∼ 0.78 and 0.5 < *p*_*s*_ < 1.1 where no repeating pattern of oscillations is observed. The emergence of discrete regions of higher-order oscillations that do not correlate with decreasing asymmetry parameter *Z*, as may be expected, arise due to the highly nonlinear behavior of the system, which is consistent with prior observations [31, 32].

The suppression of more complex-oscillation patterns is due to (1) the correction of contact forces from the original formulation [32] that erroneously introduced nonlinear behavior [46], (2) the addition of a fluid force due to flow curvature (see Section 2.1.1) that has the effect of providing a smooth loading function, as opposed to the discreet change in loading that is introduced by solely the BLEAP loading [28, 30], and (3) the addition of acoustic interactions.

Interestingly, the implementation of the BLAST solver (Fig 7C) has a minimal impact on the VF kinematics, producing a slight shift in the boundary between the 1: 1 and 1: 2 oscillation regimes to higher *Z* values, and introducing a small region of fractional oscillation ratios for low pressures and asymmetry parameters. The regime map produced by the BLAST-*n*(*t*) (Case 4) solver (not shown for brevity) was essentially identical to the BLAST solver (Case 3) shown in Fig 7C. These findings are important, demonstrating that even with significant alterations in the fluid loading, the kinematics appear to be only mildly influenced, although appreciable differences do arise in the acoustics, as is discussed in the following section.

### 4.2 Asymmetric flow and acoustic solver: Influence on acoustic measures

**Fig 8:**
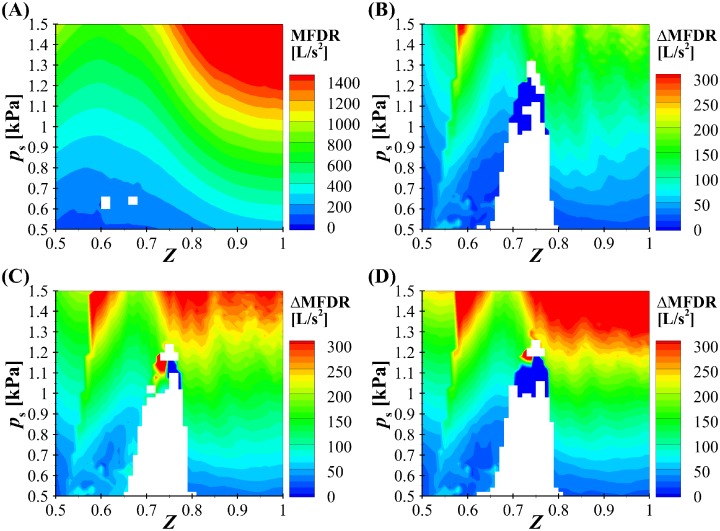
Regime map of the maximum flow declination rate (MFDR). A: Bernoulli-WRA (Case 1), B: the difference between BLEAP-WRA (Case 2) and Bernoulli-WRA (Case 1), C: the difference between BLAST (Case 3) and Bernoulli-WRA (Case 1), and D: the difference between BLAST-*n*(*t*) (Case 4) and Bernoulli-WRA (Case 1). The white color corresponds to the regions where no repeating pattern of oscillation was found in Fig 7.

**Fig 9:**
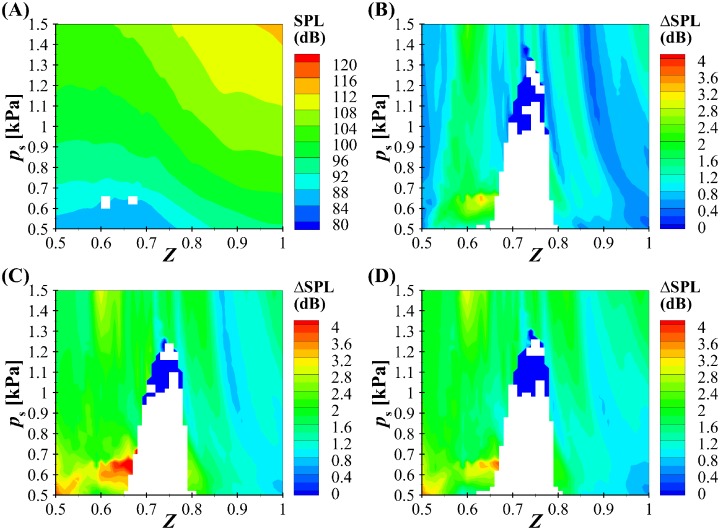
Regime map of the radiated sound pressure level (SPL). A: Bernoulli-WRA (Case 1), B: the difference between BLEAP-WRA (Case 2) and Bernoulli-WRA (Case 1), C: the difference between BLAST (Case 3) and Bernoulli-WRA (Case 1), and D: the difference between BLAST-*n*(*t*) (Case 4) and Bernoulli-WRA (Case 1). The white color corresponds to the regions where no repeating pattern of oscillation was found in Fig 7.

Interestingly, despite the minimal influence on the kinematics of the VF oscillations, the BLAST (case 3) solver does have a significant impact on acoustical measures of interest. Fig 8 presents contour plots of the maximum flow declination rate (MFDR) as a function of subglottal pressure (*p*_*s*_) and asymmetry parameter (*Z*). Fig 8A is a contour plot of the maximum MFDR for the Bernoulli-WRA (Case 1) investigations, whereas Fig 8B through Fig 8D present the differences in MFDR, relative to the BLEAP-WRA solution (Case 1), for the BLEAP-WRA (Case 2), BLAST (Case3), and the BLAST-*n*(*t*) (Case 4) formulations, respectively. In all cases, data have been suppressed in regions for which no repeating oscillatory pattern was found. Fig 9 presents contour plots of the radiated sound pressure level (SPL), with subplots A, B, C, and D calculated the same as in Fig 8. MFDR is computed as the maximum negative slope of the volumetric flow rate. The radiated SPL is computed at a distance of 15 cm from the mouth by computing the root-mean-squared value of the pressure and its logarithmic equivalent referenced to 20 *μ*Pa.

For both the MFDR and SPL contour plots in Figs 8 and 9 the highest magnitude of MFDR and SPL for BLEAP-WRA (Case 2) is, not surprisingly, found for symmetrically tensioned folds and increasing subglottal pressure. The inclusion of asymmetric fluid formation in the formulation of the BLEAP-WRA and BLAST acoustic solvers creates a significant influence on both the MFDR and SPL levels. The inclusion of asymmetric flow in the BLEAP-WRA formulation increases the MFDR by as high as 200 L/s^2^. The BLAST solution has an even greater impact, with differences in MFDR reaching greater than 300 L/s^2^ for high subglottal pressures, and 200 L/s^2^ for subglottal pressures in the range of normal speech production. Allowing for a variable glottal angle value of *n* in Fig 8D produces an even greater deviation from the Bernoulli-WRA flow solver at higher subglottal pressures, although interestingly, for low subglottal pressures (*p*_*s*_ < 0.6) the difference in the MFDR is decreased when compared with the influence of the BLAST solution (Fig 8B).

The radiated SPL for the Bernoulli-WRA (Case 1) solver (Fig 9A) is similar to the behavior of the MFDR plot. Changes in SPL due to the inclusion of asymmetric fluid loading through the BLEAP-WRA solution in Fig 9B (Case 2) introduces up to 3 dB differences. By incorporating flow asymmetries in the acoustic solution via the BLAST formulation in Fig 9C (Case 3), differences up to 4 dB are observed. Implementing a variable value for the glottal angle value *n* with the BLAST-*n*(*t*) solver (Case 4) (Fig 9D) has a very minor influence on the SPL level, when compared with the BLAST solver presented in Fig 9C. These findings are of importance, as 1 dB is a physiologically-discernible sound pressure level difference at comfortable loudness.

To enable comparison of acoustic outputs across all 4 cases, Table 2 compares the impact of fundamental frequency (*F*0), SPL, MFDR, and spectral tilt (*S*_tilt_) for the case of *p*_*s*_ = 1.3 kPa and *Z* = 1.0. While incremental changes in the fluid and acoustic formulation (moving from one case to the next) introduces modest changes in the acoustic parameters, the cumulative effect of modeling the flow and acoustic propagation as both symmetric (case 1) versus both asymmetric with variable glottal angle value *n* (case 4) introduces significant changes in the SPL, which increases by (> 1 dB), and the MFDR, which increases by (> 3000 L/s^2^(18%)). Only small changes are observed in the spectral tilt, with no impact on the fundamental frequency.

**Table 2:** Impact of the Bernoulli-WRA flow and acoustic solver (Bernoulli-WRA (Case 1)), BLEAP flow solver and WRA acoustic solver (BLEAP-WRA (Case 2)), BLAST fluid and acoustic solver with constant exponent *n* (BLAST (Case 3)), and BLAST fluid and acoustic solver with variable exponent *n* (BLAST-*n*(*t*) (Case 4)) and their effect on fundamental frequency (*F*0), sound pressure level (SPL), maximum flow declination rate (MFDR) and spectral tilt (*S*_tilt_) for *p*_*s*_ = 1.3 kPa and *Z* = 1.0.

Variable	Bernoulli-WRA (Case 1)	BLEAP-WRA (Case 2)	BLAST (Case 3)	BLAST-*n*(*t*) (Case 4)
*F*0 (Hz)	145.3	145.3	145.3	145.3
SPL (dB)	110.8	112.0	112.3	112.5
MFDR (L/s^2^)	1, 653.7	1, 812.9	1, 899.5	1, 957.6
*S*_tilt_ (dB/oct)	−9.6	−9.6	−9.7	−9.7

More detailed comparisons are also investigated for a case of asymmetrically tensioned speech, as presented in Table 3, where *p*_*s*_ = 0.65 kPa and *Z* = 0.5. While only modest changes in the acoustic measures are observed with the introduction of the asymmetric fluid solver (BLEAP-WRA (case 2)) dramatic changes arise when flow asymmetry is considered in the acoustic solver as well (BLAST (case 3)). The allowance for a time-varying glottal angle value of *n* (BLAST-*n*(*t*) (case 4)) causes minimal changes when compared with the constant *n* values investigations of case 3. Over the spectrum of solvers investigated, appreciable changes are observed as the SPL varies by 2.8 dB, the MFDR by 23.6 L/s^2^(15%), and the spectral tilt by 2.4 dB/Oct.

**Table 3:** Impact of Bernoulli-WRA (Case 1), BLEAP-WRA (Case 2), BLAST(Case 3), and BLAST-*n*(*t*) (Case 4), and their effect on fundamental frequency (*F*0), sound pressure level (SPL), maximum flow declination rate (MFDR) and spectral tilt (*S*_tilt_) for *p*_*s*_ = 0.65 kPa and *Z* = 0.5.

Variable	Bernoulli-WRA (Case 1)	BLEAP-WRA (Case 2)	BLAST (Case 3)	BLAST-*n*(*t*) (Case 4)
*F*0 (Hz)	68.4	68.4	68.4	68.4
SPL (dB)	90.2	90.9	93.3	93.0
MFDR (L/s^2^)	153.3	167.2	181.3	176.9
*S*_tilt_ (dB/oct)	−16.1	−14.4	−13.7	−14.1

These findings are consistent with clinical [8] and computational [10] investigations that have similarly observed that significant acoustic variations can arise when there are only modest asymmetries in the kinematics. The current formulation captures this acoustic variance through implementation of fluid and acoustic solvers that consider asymmetric flow development (even when the kinematic motion is symmetric). These observations become particularly salient considering that, firstly, asymmetric flow is more predominant in the presence of asymmetric glottal passages [16, 17, 23], which recent studies have shown are quite common. Secondly, current clinical fluid and acoustic measurement methods are not able to measure the intraglottal and supraglottal velocity fields with a sufficient level of detail to resolve the complex interactions that arise due to flow interactions with supraglottal structures [47, 48].

In tandem with the current study, these observations suggest that more refined fluid models may be necessary to accurately resolve the acoustic intricacies of both normal and pathological voiced speech production, and to understand why patient-specific variations in clinical measures arise; namely, the lack of correlation between the kinematics and the acoustics. With this consideration, care should also be taken to recognize that the current formulation relies upon a number of assumptions about the flow behavior, which have been carefully identified; this includes assumptions that the glottal flow can be discretized into two flow regimes (regions (1) and (2) of Fig 1), and that the pressure loading arising from the attached glottal wall jet is well approximated by a uniform flow over a translating and rotating plate, as the viscous stresses in the core of the glottal jet are negligible, as discussed inSection 2.1.2. In addition, higher order effects such as vortex shedding and transition to turbulence [18] are neglected. Finally, the assumption of one-dimensional plane wave propagation and that the acoustics are modeled as solely a dipole sound source, while capturing the primary physics, neglects monopole and quadrupole contributions, which may also occur during voiced speech production [49].

## 5 Conclusions

An acoustic glottal source model based on the BLEAP flow solver [28] was proposed to investigate the impact of higher-order glottal flow asymmetries on acoustic measures. Comparisons between the standard symmetric Bernoulli-WRA (Case 1) solver, and the asymmetric BLAST (Case 3) solver revealed the BLAST increased the radiated sound pressure level and maximum flow declination rate, while decreasing the spectral tilt.

In comparing the impact of modeling the fluid and acoustic solutions using a symmetric, versus asymmetric formulation with a time-varying glottal angle value *n*, it was found that the VF dynamics, investigated by exploring the ratios of oscillation between the left and right VFs as a function of tissue asymmetry, were only marginally influenced across a large range of subglottal pressures and asymmetry parameters. Nevertheless, despite the small changes in the dynamics, there were appreciable changes observed in the acoustic output, with the SPL, MFDR, and spectral tilt changing by up to 2.8 dB, 303.9 L/s^2^, and 2.0 dB/oct, respectively. These variations were even more exacerbated for instances of irregularly-tensioned VF properties that mimic unilateral paresis and paralysis, where the proposed model introduces significant acoustic and aerodynamic differences without affecting the VF kinematics.

These findings highlight how effects arising from glottal flow asymmetries that are commonly neglected in both clinical and reduced-order modeling investigations may play an important role in the acoustics of voiced speech production, thereby yielding insight into the unexplained variance in acoustic measures that has been observed clinically [8, 50].

The manner in which the acoustical solution was developed facilitates easy implementation into existing reduced-order VF models, making it broadly applicable.

## Supporting information

S1 Dataset Data for Fig 4. (DAT)

S2 Dataset Data for Fig 5. (DAT)

S3 Dataset Data for Fig 6. (DAT)

S4 Dataset Data for Figs 7–9 corresponding to the Bernoulli solver (case 1). The data contained herein is plotted in Figs 7A, 8A and 9A.
(DAT)

S5 Dataset Data for Figs 7–9 corresponding to the BLEAP-WRA solver (case 2). The data contained herein is plotted in Fig 7B, and in Figs 8B and 9B by computing the difference between the S5 Dataset and S4 Dataset.
(DAT)

S6 Dataset Data for Figs 7–9 corresponding to the BLAST solver (case 3). The data contained herein is plotted in Fig 7C, and in Figs 8C and 9C by computing the difference between the S6 Dataset and S4 Dataset.
(DAT)

S7 Dataset Data for Figs 7–9 corresponding to the BLAST-*n*(*t*) solver (case 4). The data contained herein is plotted in Figs 8C and 9C by computing the difference between the S7 Dataset and S4 Dataset.
(DAT)
